# Supplementary material for: Proclivity of nervous system preservation in Cambrian Burgess Shale-type deposits
Source: Proc Biol Sci. 2019 Dec 11;286(1917):20192370. doi: 10.1098/rspb.2019.2370 (PMC6939931; doi:10.1098/rspb.2019.2370)
Supplement: Supplementary Figures and Table [file rspb20192370supp1.docx]

Supplementary Information for

**Proclivity of nervous system preservation in Cambrian Burgess Shale-type deposits**

Javier Ortega-Hernández^1,*^

Rudy Lerosey-Aubril^1,*^

Stephen Pates^1^

^1^Museum of Comparative Zoology and Department of Organismic and Evolutionary Biology, Harvard University, 26 Oxford Street, Cambridge, MA 02138, USA.

Correspondence: [jortegahernandez@fas.harvard.edu](mailto:jortegahernandez@fas.harvard.edu); rudy_lerosey@fas.harvard.edu

**This PDF file includes:**

Figures S1 and S2

Table S1

SI References

**
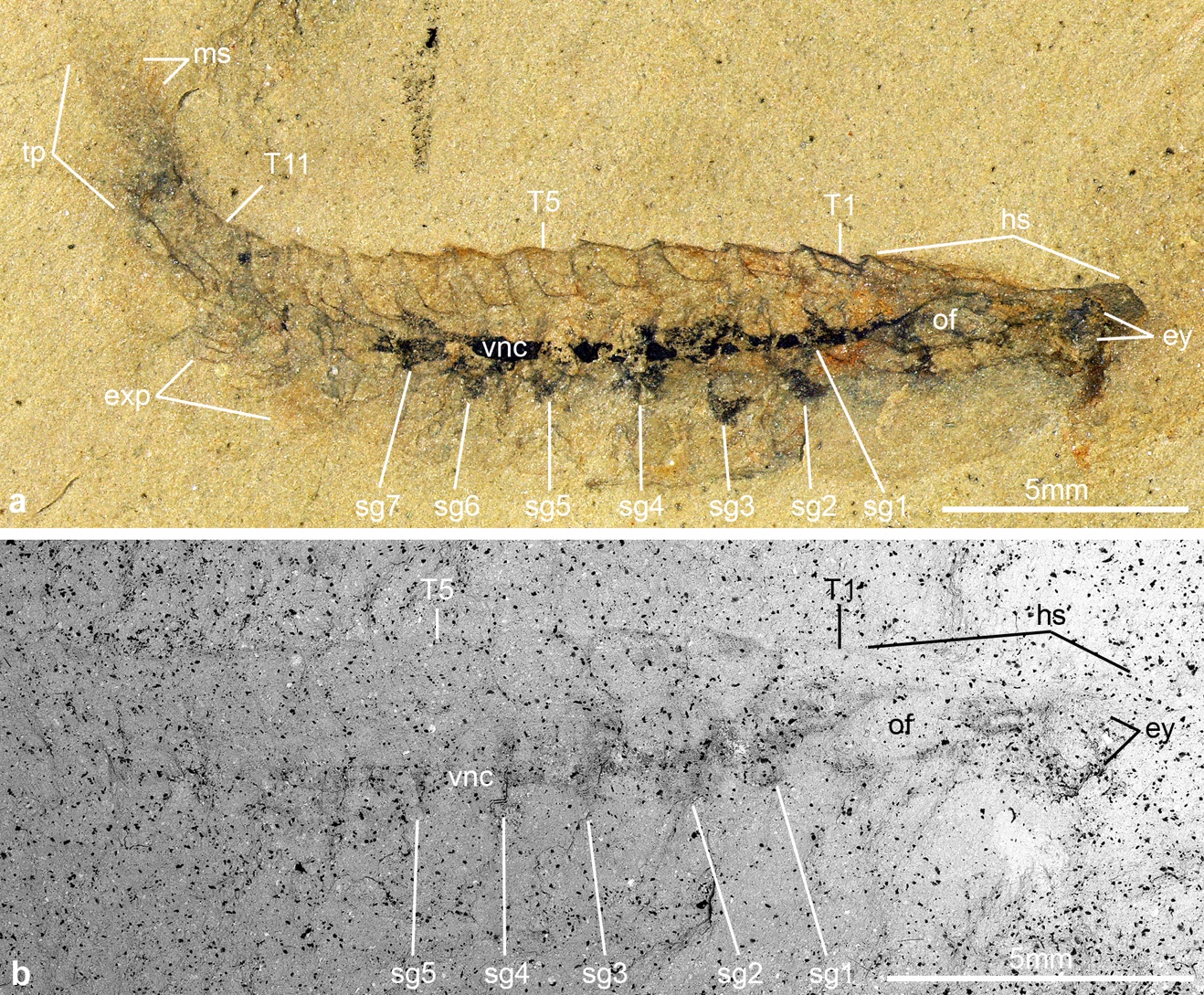
**

**Fig. S1. Compositional analysis of *Alalcomenaeus* from the Wuliuan Pioche Formation. a.** MCZ IP-197956a part photographed under reflected light. **b.** MCZ IP-197956b imaged with backscatter electron microscopy. Abbreviations: ey, stalked eye; exp, trunk exopod; hs, head shield; of, oesophageal foramen; sg*n*, segmental ganglia; T*n*, trunk tergites; vnc, ventral nerve cord.


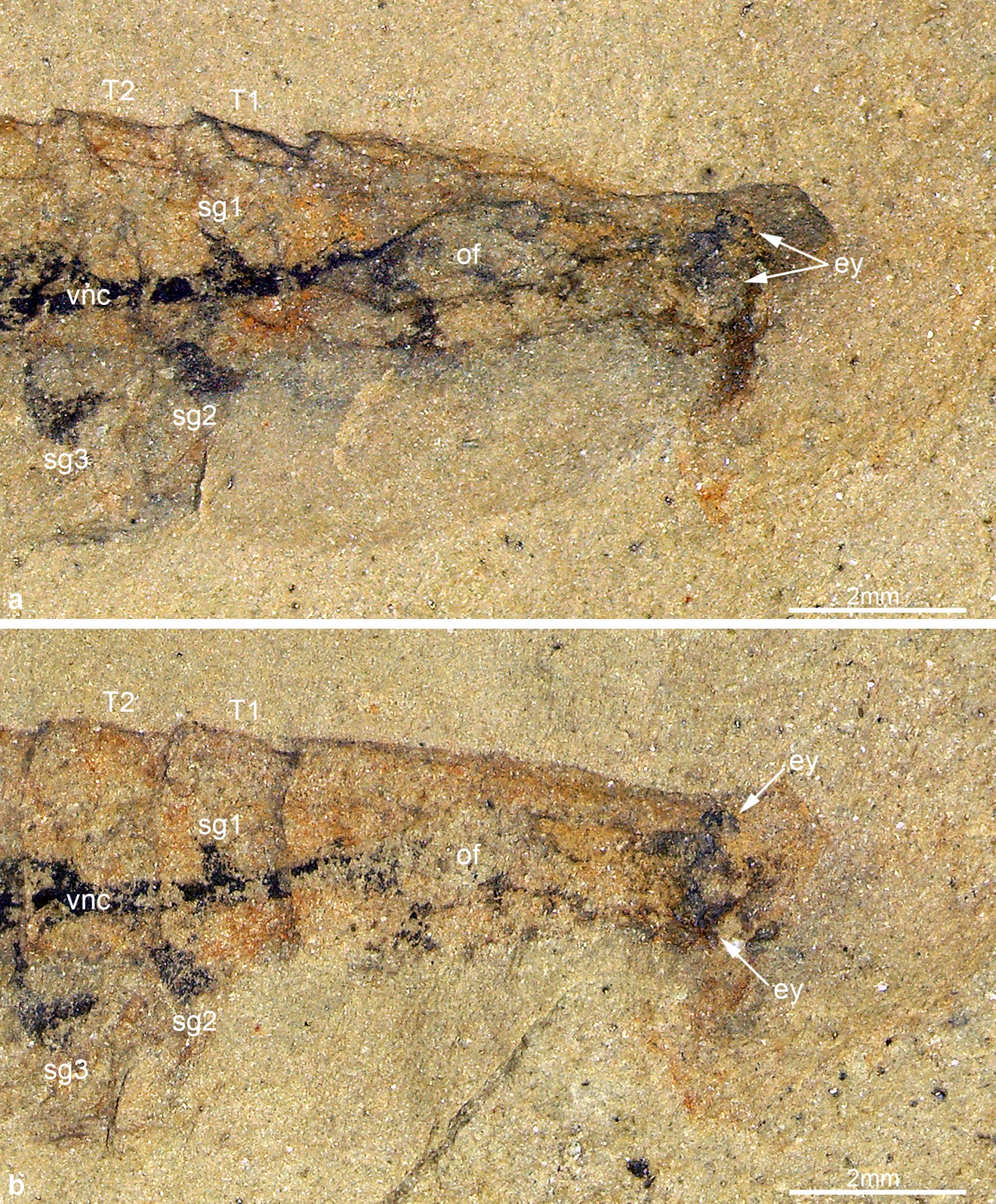


**Fig. S2. Detail of the head organization in *Alalcomenaeus* from the Stage 4 Pioche Formation. a.** MCZ IP-197956a part, showing preservation of adaxial pair of stalked ventral eyes. **b.** MCZ IP-197956b counterpart, showing preservation of abaxial pair of stalked ventral eyes. Note that the photograph orientation is mirrored to ease comparison with part. Abbreviations: ey, stalked eye; of, oesophageal foramen; sg*n*, segmental ganglia; T*n*, trunk tergites; vnc, ventral nerve cord.

| **Table S1. Global occurrence of central nervous system (CNS) preservation in Cambrian Burgess Shale-type deposits.** Asterisk denotes availability of published elemental data.  ‡ denotes observable neuroanatomical features, but not detectable through elemental analyses. | | | |
| --- | --- | --- | --- |
| **Taxon** | **Age and locality** | **CNS preservation** | **Reference** |
| *Kerygmachela kierkegaardi* | Cambrian (Stage 3)  Sirius Passet  North Greenland | dorsal brain  optic nerve | Park et al. 2018* |
| *Fuxianhuia protensa* | Cambrian (Stage 3)  Chengjiang biota  South China | dorsal brain  optic neuropils  optic nerves | Ma et al. 2012*  Ma et al. 2015* |
| *Alalcomenaeus* sp. | Cambrian (Stage 3)  Chengjiang biota  South China | dorsal brain  optic neuropils  optic nerves  oesophageal foramen  ventral nerve cord  segmental nerves  posterior connectives | Tanaka et al. 2013* |
| *Lyrarapax unguispinus* | Cambrian (Stage 3)  Chengjiang biota  South China | dorsal brain  optic neuropils  optic nerves | Cong et al. 2014* |
| *Chengjiangocaris kunmingensis* | Cambrian (Stage 3)  Xiaoshiba biota  South China | ventral nerve cord  segmental nerves | Yang et al. 2016‡ |
| *Waptia fieldensis* | Cambrian (Wuliuan)  Burgess Shale  British Columbia | dorsal brain  optic neuropils  ventral nerve cord? | Strausfeld 2011, 2016  Vannier et al. 2018* |
| *Mollisonia plenovenatrix* | Cambrian (Wuliuan)  Marble Canyon  British Columbia | optic neuropils  optic nerves | Aria & Caron 2019* |

| **Table S1 continued.** | | | |
| --- | --- | --- | --- |
| **Taxon** | **Age and locality** | **CNS preservation** | **Reference** |
| *Ottoia prolifica* | Cambrian (Wuliuan)  Burgess Shale  British Columbia | ventral nerve cord | Edgecombe et al. 2015 |
| *Helmetia expansa* | Cambrian (Wuliuan)  Burgess Shale  British Columbia | optic nerves | Ortega-Hernández 2015‡ |
| *Odaraia alata* | Cambrian (Wuliuan)  Burgess Shale  British Columbia | dorsal brain  optic nerves | Ortega-Hernández 2015‡ |
| *Canadia spinosa* | Cambrian (Wuliuan)  Burgess Shale  British Columbia | terminal brain  ventral nerve cord  parapodial nerves | Parry & Caron 2019* |
| *Pikaia gracilens* | Cambrian (Wuliuan)  Burgess Shale  British Columbia | dorsal ?nerve cord | Conway-Morris & Caron 2012* |
| *Alalcomenaeus* sp. | Cambrian (Stage 4)  Pioche Formation  Utah, USA | dorsal brain  optic nerves  oesophageal foramen  ventral nerve cord  segmental nerves  posterior connectives | this study* |
| *Alalcomenaeus* sp. | Cambrian (Drumian)  Marjum Formation  Utah, USA | dorsal brain  optic nerves  oesophageal foramen?  ventral nerve cord  segmental nerves | this study‡ |

**SI References**

- Aria C, Caron JB. 2019. A middle Cambrian arthropod with chelicerae and proto-book gills. *Nature*, **573**, 586-589.
- Cong P, Ma X, Hou X, Edgecombe GD, Strausfeld NJ. 2014. Brain structure resolves the segmental affinity of anomalocaridid appendages. *Nature* **513**, 538-542.
- Conway-Morris S., Caron JB. 2012. *Pikaia gracilens* Walcott, a stem‐group chordate from the Middle Cambrian of British Columbia. *Biological Reviews* **87**, 480-512.
- Edgecombe GD, Ma X, Strausfeld NJ. 2015. Unlocking the early fossil record of the arthropod central nervous system. *Philosophical Transactions of the Royal Society B: Biological Sciences* **370**, 20150038.
- Ma X, Hou X, Edgecombe GD, Strausfeld NJ. 2012. Complex brain and optic lobes in an early Cambrian arthropod. *Nature* **490**, 258-261.
- Ma X, Edgecombe GD, Hou X, Goral T, Strausfeld NJ. 2015. Preservational pathways of corresponding brains of a Cambrian euarthropod. *Current Biology* **25**, 2969-2975.
- Ortega-Hernández J. 2015. Homology of head sclerites in Burgess Shale euarthropods. *Current Biology* **25**, 1625-1631.
- Park TYS, Kihm JH, Woo J, Park C, Lee WY, Smith MP, Harper DA, Young F, Nielsen AT, Vinther J, 2018. Brain and eyes of *Kerygmachela* reveal protocerebral ancestry of the panarthropod head. *Nature communications* **9**, 1019.
- Parry L, Caron JB. 2019. *Canadia spinosa* and the early evolution of the annelid nervous system. *Science Advances* **5**, eaax5858.
- Strausfeld NJ, 2011. Some observations on the sensory organization of the crustaceomorph *Waptia* *fieldensis* Walcott. *Palaeontographica Canadiana* **31**, 157-168.
- Strausfeld NJ. 2016. *Waptia* revisited: intimations of behaviors. *Arthropod Structure & Development* **45**, 173-184.
- Tanaka G, Hou X, Ma X, Edgecombe GD, Strausfeld NJ, 2013. Chelicerate neural ground pattern in a Cambrian great appendage arthropod. *Nature* **502**, 364-367.
- Vannier J, Aria C, Taylor RS, Caron JB, 2018. *Waptia fieldensis* Walcott, a mandibulate arthropod from the middle Cambrian Burgess Shale. *Royal Society Open Science* **5**, p.172206.
- Yang J, Ortega-Hernández J, Butterfield NJ, Liu Y, Boyan GS, Hou JB, Lan T, Zhang XG. 2016. Fuxianhuiid ventral nerve cord and early nervous system evolution in Panarthropoda. *Proceedings of the National Academy of Sciences*, **113**, 2988-2993.
